# The intrinsic preference of guanosine bases for cleavage-facilitating interactions with phosphodiester moieties in RNA anions revealed by base modifications and mass spectrometry

**DOI:** 10.1093/nar/gkaf494

**Published:** 2025-06-16

**Authors:** Anna Ploner, Christoph Mitteregger, Heidelinde Glasner, Raphael Bereiter, Ronald Micura, Kathrin Breuker

**Affiliations:** Institute of Organic Chemistry and Center for Molecular Biosciences Innsbruck (CMBI), University of Innsbruck, Innrain 80–82, 6020 Innsbruck, Austria; Institute of Organic Chemistry and Center for Molecular Biosciences Innsbruck (CMBI), University of Innsbruck, Innrain 80–82, 6020 Innsbruck, Austria; Institute of Organic Chemistry and Center for Molecular Biosciences Innsbruck (CMBI), University of Innsbruck, Innrain 80–82, 6020 Innsbruck, Austria; Institute of Organic Chemistry and Center for Molecular Biosciences Innsbruck (CMBI), University of Innsbruck, Innrain 80–82, 6020 Innsbruck, Austria; Institute of Organic Chemistry and Center for Molecular Biosciences Innsbruck (CMBI), University of Innsbruck, Innrain 80–82, 6020 Innsbruck, Austria; Institute of Organic Chemistry and Center for Molecular Biosciences Innsbruck (CMBI), University of Innsbruck, Innrain 80–82, 6020 Innsbruck, Austria

## Abstract

Spontaneous backbone cleavage of ribonucleic acids (RNAs) in basic aqueous solution has a preference for the 5′ side of guanosine. This phenomenon can also be observed in fully desolvated RNA (M − *n*H)^*n*−^ ions subjected to vibrational activation by low-energy collisionally activated dissociation. However, the underlying chemical mechanism of the cleavage reaction has so far remained elusive. Using RNA with site-specific deaza (c^1^G, c^3^G, c^7^G) or methyl (m^2^G, m^2^_2_G) modifications and RNA with guanosine to inosine substitution in a comparative study, we show here that preferential cleavage is due to bidentate interactions of guanosine bases with the nonbridging oxygens of phosphodiester moieties on their 5′ side. The unimolecular chemistry involved in the RNA cleavage reaction on the 5′ side of guanosine may help to understand the evolution of catalytic strategies employed by self-cleaving ribozymes.

## Graphical Abstract

**Graphical Abstract:**
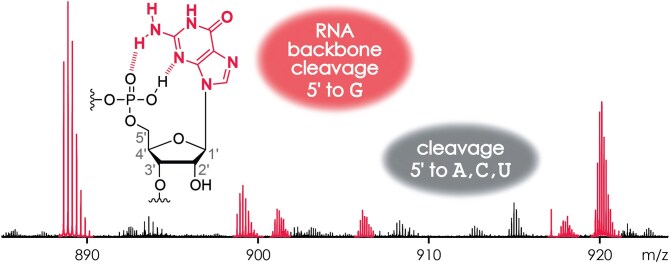

## Introduction

**Scheme 1.**
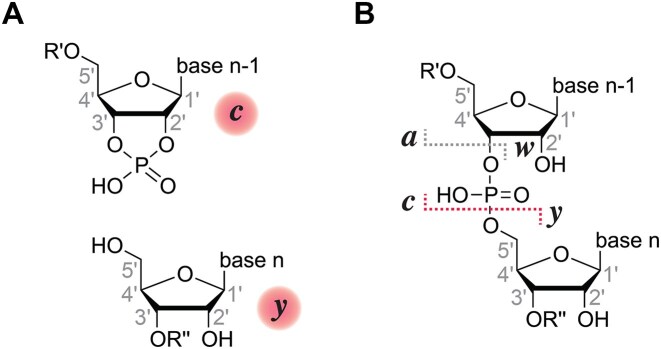
The chemical structures of 2′,3′ cyclic phosphate and 5′-OH products from RNA cleavage by small self-cleaving ribozymes [1], ribonucleases [50], and acid- or base-catalyzed hydrolysis [8] in solution (**A**) correspond to the *c* and *y* fragments (**B**) from collisionally activated dissociation (CAD) of gaseous RNA (M + *n*H)^*n*+^ and (M − *n*H)^*n*−^ ions in MS experiments [51].

Ribonucleic acids (RNAs) have important functions beyond storing and transmitting genetic information, including the catalysis of chemical reactions in living systems as ribozymes [1]. The most prominent reaction catalyzed by naturally occurring ribozymes is peptide bond formation on the ribosome [2], but the reactions most commonly catalyzed by them are phosphodiester backbone bond cleavage and ligation [3]. Phosphodiester backbone bond cleaving ribozymes are divided into two different classes, depending on whether they catalyze the formation of products with 5′-phosphate and 3′-hydroxyl or 2′,3′-cyclic phosphate and 5′-hydroxyl termini [4]. The large number of small self-cleaving ribozymes belonging to the latter class cleave their own backbone [5–7]. The proposed mechanisms for RNA phosphodiester backbone bond cleavage into products with 2′,3′-cyclic phosphate and 5′-hydroxyl termini (Scheme 1A) invariably involve nucleophilic attack of a ribose 2′ oxygen on the phosphorus of the adjacent phosphodiester moiety, regardless of whether the reaction occurs in solution [8] or in the gas phase [9, 10], and whether or not it is catalyzed by protein enzymes or ribozymes [4]. Importantly, the active sites of different classes of small self-cleaving ribozymes can use a combination of four different strategies to catalyze phosphodiester backbone bond cleavage, as classified by Breaker and coworkers [11, 12]. These are α catalysis (positioning of the ribose 2′ oxygen in line with the phosphorus and the 5′ oxygen of the phosphodiester moiety), β catalysis (neutralization of negative charge on the nonbridging oxygen of the phosphodiester moiety), γ catalysis (deprotonation of the 2′ hydroxyl group), and δ catalysis (neutralization of the developing negative charge on the 5′ oxygen). As reviewed by Bevilacqua and Yajima, ribozymes can use their nucleobases directly in chemical catalysis [13]. In this context, X-ray crystallography and atomic mutagenesis studies provided strong evidence for specific, catalytic interactions of nucleobases with the phosphodiester moiety in the active sites of different ribozymes [1, 14, 15]. Interestingly, the extent to which each strategy contributes to catalysis has only recently been elucidated, and this scaling has been found to be different for different ribozymes [14, 15].

RNA phosphodiester backbone cleavage can also be achieved by low-energy CAD of fully desolvated, gaseous RNA (M + *n*H)^*n*+^ and (M − *n*H)^*n*−^ ions from electrospray ionization (ESI) in mass spectrometry (MS) experiments [16], where the products with 2′,3′-cyclic phosphate and 5′-hydroxyl termini are referred to as *c* and *y* fragments (Scheme 1B). Reminiscent of β catalysis, the energy required for backbone cleavage in RNA (M + *n*H)^*n*+^ and (M − *n*H)^*n*−^ ions, in which the backbone is uncharged and partially deprotonated, respectively, is much lower for the RNA (M + *n*H)^*n*+^ ions, as we have shown in a previous study [9]. In the same study, we found that adenosine and guanosine can facilitate cleavage of the phosphodiester backbone bond on their 5′ side in CAD of RNA (M + *n*H)^*n*+^ and (M − *n*H)^n−^ ions, respectively [9]. In combination with atomic mutagenesis, further MS investigation of RNA (M + *n*H)^*n*+^ ions provided evidence that preferential cleavage on the 5′ side of adenosine results from N3 protonation and the formation of an ionic hydrogen bond between protonated adenosine and a nonbridging oxygen of the adjacent phosphodiester moiety [10]. The effect of guanosine on RNA backbone cleavage in CAD of (M − *n*H)^*n*−^ ions, which we address here, turned out to be more difficult to understand as it was more pronounced in some CAD spectra than in others for no immediately obvious reason. Again building on the power of atomic mutagenesis, in particular by using RNA with site-specific deaza (c^1^G, c^3^G, c^7^G) and also methyl (m^2^G, m^2^_2_G) modifications and guanosine to inosine (I) substitution in CAD of (M − *n*H)^*n*−^ ions, we have now obtained strong evidence for cleavage-facilitating interactions of guanosine bases with both nonbridging oxygens of the phosphodiester moieties on their 5′ side. A facilitating effect of guanosine on RNA hydrolysis was also observed in solutions at high pH, in agreement with previous results from the Breaker laboratory [17]. We discuss our gas-phase and solution data on RNA phosphodiester backbone bond cleavage in the context of the catalytic strategies used by small self-cleaving ribozymes.

## Materials and methods

### RNA synthesis and preparation of solutions for ESI

Deazaguanosine (c^1^G, c^3^G, c^7^G) phosphoramidites [15, 18], mono- (m^2^G) and dimethylated (m^2^_2_G) guanosine phosphoramidites [19], and inosine phosphoramidites [20] were prepared by chemical synthesis. RNAs **1**–**12** (Table 1) were prepared by solid-phase synthesis [21, 22] and purified by anion exchange chromatography. Fractions containing RNA were diluted with 0.1 M triethylammonium bicarbonate in H_2_O, loaded on a C18 SepPak Plus cartridge (Waters/Millipore), washed with H_2_O, and eluted with 1:1 acetonitrile/H_2_O. For further desalting of RNAs **1**–**9**, 400 μl of 100 mM ammonium acetate solution in H_2_O was added to 100 μl RNA solution (1–10 nmol in H_2_O) and concentrated to ∼100 μl using Vivaspin 500 centrifugal concentrators (Sartorius, Germany, PES membrane, MWCO 3000). The process was repeated 5–10 times, followed by six to seven cycles of concentration and dilution with H_2_O (RNA **1**–**6**) or 1:1 H_2_O/CH_3_OH (RNAs **7**–**9**). RNA concentration was determined by UV absorption at 260 nm using a NanoPhotometer (Implen, Germany), for which nucleotide extinction coefficients from [23] were used. All nucleobases with modifications were treated as unmodified nucleobases assuming that they have a negligible effect on the extinction coefficient of the RNAs.

**Table 1.** RNA studied

RNA	Sequence^a^	nt	*M*_measured_^b^	*M*_calculated_^b^
**1**	ACCCG CAAGG CCGAC GGC	18	5764.871	5764.869
**2**	ACCC**c^3^G** CAAGG CCGAC GGC	18	5763.877	5763.874
**3**	ACCC**c^1^G** CAAGG CCGAC GGC	18	5763.872	5763.874
**4**	ACCC**I** CAAGG CCGAC GGC	18	5749.857	5749.858
**5**	ACCC**I** CAA**I**G CC**I**AC GGC	18	5719.836	5719.836
**6**	AUCUG CUUGC CCAUC GGGGC CGCGG AU	27	8612.129	8612.152
**7**	AUCU**m^2^G** CUUGC CCAUC GGGGC CGCGG AU	27	8626.139	8626.168
**8**	AUCUG CUUGC CCAUC G**m^2^G**GGC CGCGG AU	27	8626.154	8626.168
**9**	AUCUG CUUGC CCAUC GGGGC C**m^2^_2_G**CGG AU	27	8640.182	8640.183
**10**	GGCUA GCC	8	2523.389	2523.388
**11**	GGCUA **c^3^G**CC	8	2522.392	2522.393
**12**	GGCUA **c^7^G**CC	8	2522.392	2522.393

^a^From 5′-OH- to 3′-OH-terminus.

^b^ *M* refers to monoisotopic mass in Da.

Sequences of the canonical, deaza- or methyl-modified, and guanosine to inosine substituted 18-nt, 27-nt, and 8-nt RNAs used in this study are listed in Table 1. The sequence of the 18-nt RNAs **1**–**5** is derived from the twister-sister ribozyme [24], and calculations using the Vienna RNAfold Server (http://rna.tbi.univie.ac.at) predict that RNA **1** can only partially fold in solution into a very weak (−8 kJ/mol) stem-loop structure consisting of only three base pairs and a rather short GAC loop. The sequence of the 27-nt RNAs **6**–**9** corresponds to spanning repeat 8 of XIST RNA [25], and RNA **6** has a predicted hairpin structure with an internal loop (−57 kJ/mol). The palindromic sequence of the 8-nt RNAs **10**–**12** was previously designed for thermodynamic studies [15], and has no predicted secondary structure as single-stranded RNA. In the solutions for ESI, we used low RNA concentrations to prevent the formation of double stranded helices (RNAs **10**–**12**) and denaturing conditions (50% CH_3_OH at high pH) to minimize the formation of secondary structure.

### RNA hydrolysis

For hydrolysis experiments, two solutions of RNA **1** in H_2_O (18 MΩ cm) were prepared, one at pH 13.0 (200 μl, 5 μM RNA **1**, 100 mM piperidine) and the other at pH 9.0 (200 μl, 2 μM RNA **1**, 4 mM piperidine), and immediately placed into a thermomixer (Eppendorf) at 37°C and 80°C, respectively. Aliquots of the solutions were removed after the time intervals indicated and diluted to 0.5–1.0 μM RNA **1** in 1:1 H_2_O/CH_3_OH for ESI MS to determine the site-specific extent of hydrolysis. Only hydrolysis products containing the original 5′ or 3′ terminus were included in the calculation of site-specific yields, as hydrolysis products from multiple backbone cleavage were generally low in abundance and the assignment of many of these products was not unambiguous.

### Mass spectrometry

Experiments were performed on a 7- or a 12-T Fourier transform ion cyclotron resonance (FT-ICR) mass spectrometer (Bruker, Austria), both equipped with an ESI source and a collision cell floated with Ar gas for vibrational activation by CAD. RNA (M − *n*H)^*n*−^ ions were electrosprayed (flow rate 1.5 μl/min) from 0.5–1.0 μM solutions in 1:1 H_2_O/CH_3_OH with organic bases (10–20 mM piperidine and 0–20 mM imidazole) [26] as additives, except for the solution of RNA **1** at pH 8.0, for which 50 mM ammonium bicarbonate was used. Internal calibration for accurate mass measurements used polyethylene glycol 1000 (Sigma–Aldrich, Austria) as calibrant in ESI spectra (RNAs **6**–**9**) or *c* and *y* fragments in CAD spectra (RNAs **1**–**5** and **10**–**12**) (Table 1). Methanol was HPLC grade (Acros, Austria), H_2_O was purified to 18 MΩ cm at room temperature using a Milli-Q system (Millipore, Austria), ammonium acetate (≥99.0%, Na ≤5 mg/kg, K ≤5 mg/kg), piperidine (≥99.5%), and imidazole (≥99.5%, Na ≤50 mg/kg, K ≤50 mg/kg) were from Sigma–Aldrich (Austria), and pH values were measured using nonbleeding pH-indicator strips (Merck, Germany). The (M − *n*H)^*n*−^ ions of interest were isolated in a linear quadrupole prior to dissociation by CAD; for a more detailed description of the experimental setup for CAD, see [27]. For statistical reasons, up to 150 scans for ESI (for accurate mass and hydrolysis measurements) and 200 scans for CAD were added for each spectrum, and data reduction utilized the SNAP2 algorithm (Bruker, Austria) or FAST MS [28], a software programmed in our group by Michael Palasser, as well as manual inspection of the spectra.

## Results and discussion

**Figure 1.**
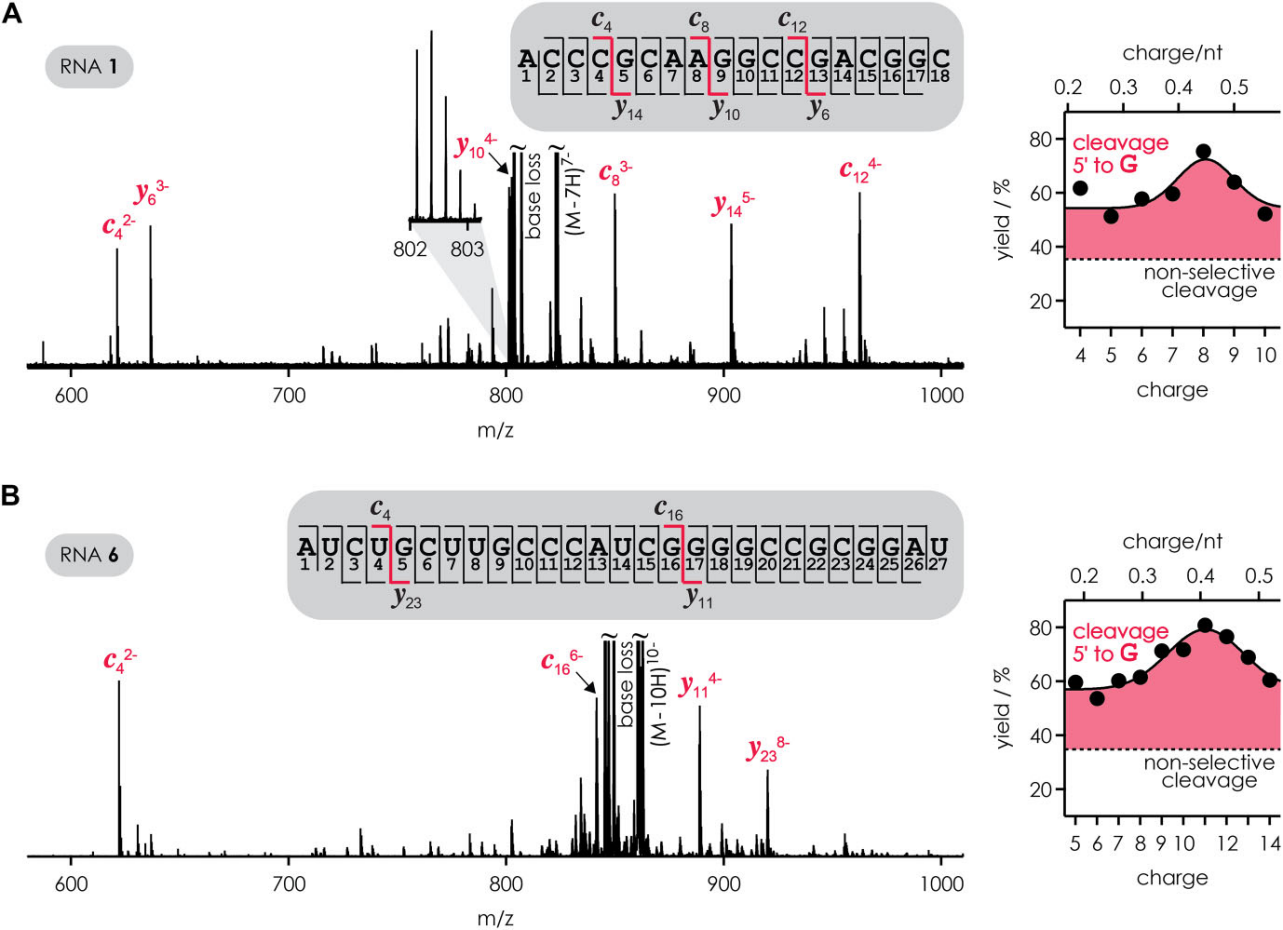
Spectra (left) from CAD of (**A**) (M − 7H)^7−^ ions of RNA **1** at 42.0 eV laboratory frame collision energy and (**B**) (M − 10H)^10−^ ions of RNA **6** at 80.3 eV provide complete sequence coverage as shown in the corresponding cleavage maps (gray boxes), but *c* and *y* fragments from phosphodiester backbone cleavage on the 5′ side of guanosine residues (red) are dominant. The yield of *c* and *y* fragments from phosphodiester backbone cleavage on the 5′ side of all guanosine residues relative to all *c* and *y* fragments (right) was found to exceed that expected for random cleavage (dashed lines) and to depend on (M − *n*H)^*n*−^ ion net charge, with maximum values at ∼0.45 (RNA **1**) and ∼0.4 (RNA **6**) charges/nt; solid lines are Gaussian fit functions meant to guide the eye.

The effect of guanosine on RNA phosphodiester backbone cleavage is illustrated in Fig. 1 with spectra from CAD of (M − 7H)^7−^ ions of RNA **1** and (M − 10H)^10−^ ions of RNA **6**. For both RNAs, cleavage products (Scheme 1B) were observed from all possible sites, i.e. the *c* and *y* fragments provided complete sequence information. However, a few fragments dominate both spectra, and all of them originate from phosphodiester backbone cleavage on the 5′ side of guanosine residues (G5: *c*_4_/*y*_14_, G9: *c*_8_/*y*_10_, G13: *c*_12_/*y*_6_ for RNA **1**; G5: *c*_4_/*y*_23_, G17: *c*_16_/*y*_11_ for RNA **6**). To quantify the overall effect of guanosine on phosphodiester backbone cleavage, we calculated the yield of *c* and *y* fragments from cleavage on the 5′ side of all guanosine residues relative to all *c* and *y* fragments. For random cleavage, we would expect a yield of 35% for both RNA **1** (6 sites on the 5′ side of G, 17 sites in total) and RNA **6** (9 sites on the 5′ side of G, 26 sites in total), but instead found 60% for the (M − 7H)^7−^ ions of RNA **1** and 72% for the (M − 10H)^10−^ ions of RNA **6**. This is consistent with previous studies in which we observed preferential cleavage on the 5′ side of guanosine in CAD of (M − *n*H)^*n*−^ [9, 29] but not (M + *n*H)^*n*+^ [10] ions of other RNAs. Here we examine the effect of guanosine on phosphodiester backbone cleavage in detail by studying the role of (M − *n*H)^*n*−^ ion charge, collision energy, the pH of the solution used for ESI, and nucleobase modifications.

To better understand preferential cleavage on the 5′ side of guanosine in RNA (M − *n*H)^*n*−^ ions, we first considered insights from our CAD studies of RNA (M + *n*H)^*n*+^ ions [9, 10]. Preferential phosphodiester backbone bond cleavage next to adenosine in CAD of RNA (M + *n*H)^*n*+^ ions is due to ionic hydrogen bonding between adenine protonated at N3 and the uncharged phosphodiester moiety on its 5′ side, which facilitates nucleophilic attack of the 2′ oxygen on the phosphorus [9, 10]. Phosphodiester backbone bonds on the 5′ side of bases other than adenine are also cleaved, but to a much lesser extent because the other bases—even if protonated—do not form ionic hydrogen bonds with phosphodiester moieties in RNA (M + *n*H)^*n*+^ ions [10]. RNA (M − *n*H)^*n*−^ ions are generally non-zwitterionic, i.e. none of the bases are protonated [9], so there is no positive charge available to potentially facilitate cleavage of phosphodiester backbone bonds. However, ESI produces gaseous (M − *n*H)^*n*−^ ions in which not every phosphodiester moiety is deprotonated; i.e. *n* is generally smaller than the number of phosphodiester moieties [26]. For example, in the (M − 5H)^5−^ and (M − 13H)^13−^ ions of RNA **6**, on average only ∼20% and 50%, respectively, of the phosphodiester moieties are deprotonated. Here we propose that hydrogen bond interactions of guanosine bases hold protons in place on the phosphodiester moieties on their 5′ side in RNA (M − *n*H)^*n*−^ ions such that phosphodiester moieties adjacent to bases other than guanosine are preferentially deprotonated. Since nucleophilic attack of the 2′ oxygen on the phosphorus of an uncharged phosphodiester moiety is favored over attack on the phosphorus of a deprotonated phosphodiester moiety (“β catalysis”), this scenario would explain the observed preferential cleavage on the 5′ side of guanosines. In addition, preferential cleavage on the 5′ side of guanosines should increase with increasing deprotonation, i.e. (M − *n*H)^*n*−^ ion charge, as the phosphodiester moieties compete for a decreasing number of protons. In support of this hypothesis, the yield of *c* and *y* fragments from cleavage on the 5′ side of guanosine not only exceeded that expected for random cleavage but increased with increasing (M − *n*H)^*n*−^ ion charge up to ∼0.45 and ∼0.4 charges/nt for RNAs **1** and **6**, respectively (Fig. 1). At higher (M − *n*H)^*n*−^ ion charge, corresponding to >0.45 and >0.4 charges/nt, the extent of phosphodiester backbone cleavage on the 5′ side of guanosine decreased again, indicating that the number of protons available for the proposed hydrogen bond interactions of guanosine bases with phosphodiester moieties on their 5′ side reaches a limit and/or that increasing Coulombic repulsion in the (M − *n*H)^*n*−^ ions leads to increasingly extended structures in which intramolecular interactions are less favorable.

**Figure 2.**
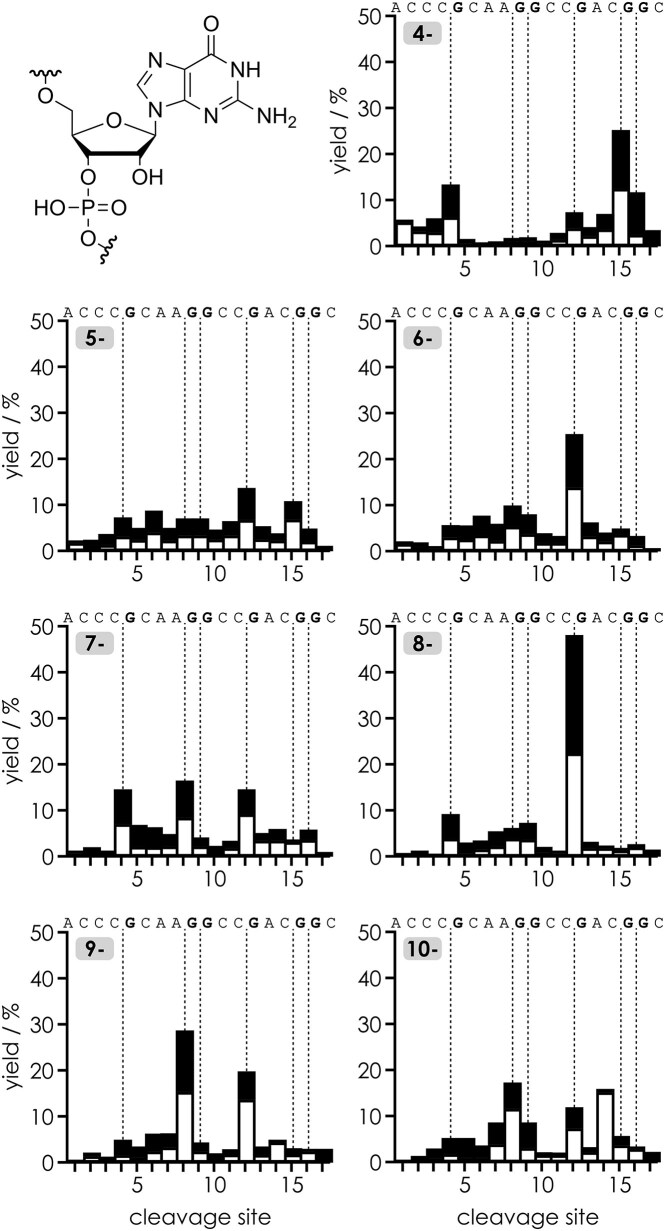
Site-specific yield of *c* (filled bars) and *y* (open bars) fragments (including those that showed nucleobase loss and relative to all *c* and *y* fragments) from CAD of (M − *n*H)^*n−*^ ions of RNA **1** for *n* = 4–10; the laboratory frame collision energy was adjusted to maximize cleavage into *c* and *y* fragments while minimizing secondary dissociation (*n* = 4: 72.0 eV, *n* = 5: 68.5 eV, *n* = 6: 64.8 eV, *n* = 7: 60.9 eV, *n* = 8: 57.6 eV, *n* = 9: 54.0 eV, *n* = 10: 50.0 eV); cleavage sites on the 5′ side of guanosine are highlighted by dashed lines.

Surprisingly, the sites of maximum cleavage, although always located on the 5′ side of guanosine residues, were different for different values of *n* as illustrated in Fig. 2 for RNA **1** and Supplementary Fig. S1 for RNA **6**, and not all guanosine residues gave rise to preferential cleavage. We attribute the charge-dependent variation in the yield of *c* and *y* fragments from cleavage on the 5′ side of guanosine to differences in the number of protons available for the proposed interactions in (M − *n*H)^*n*−^ ions with different net charge *n*. In addition, Coulombic repulsion can counteract the holding of a proton in a given position by hydrogen bonding, and Coulombic repulsion is naturally different for different *n*.

**Figure 3.**
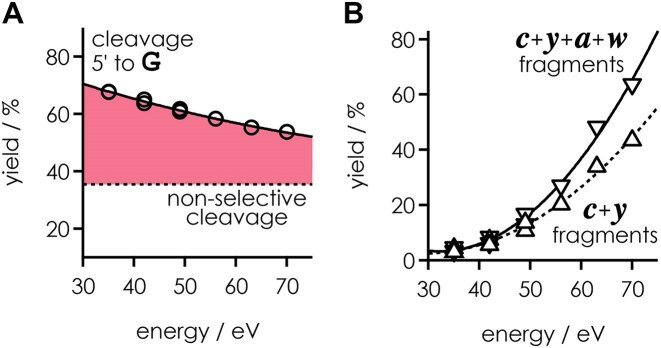
(**A**) Yield of *c* and *y* fragments from phosphodiester backbone cleavage on the 5′ side of all guanosine residues (including those that showed nucleobase loss and relative to all *c* and *y* fragments) from CAD of (M − 7H)^7−^ ions of RNA **1** and (**B**) yield of fragments from backbone cleavage relative to all species including undissociated RNA **1** and nucleobase losses from RNA **1** versus laboratory frame collision energy; lines are meant to guide the eye.

Because collisional activation can disrupt intramolecular interactions and mobilize protons [9, 30], we next investigated how the laboratory frame collision energy used for CAD affects phosphodiester backbone cleavage on the 5′ side of guanosine for the (M − 7H)^7−^ ions of RNA **1**. As shown in Fig. 3, the yield of *c* and *y* fragments from cleavage on the 5′ side of guanosine relative to all *c* and *y* fragments decreased from ∼70% at 35 eV to 55% at 70 eV, from which we conclude that the number of cleavage-facilitating interactions decreases with increasing energy. However, even at the highest energy studied, which dissociated ∼65% of the (M − 7H)^7−^ ions of RNA **1** into products from backbone cleavage (∼45% *c*, *y* and ∼20% *a*, *w* fragments), a marked effect of guanosine was still observed.

We also considered the possibility that deprotonation of guanosine bases (p*K*_a_ of N1: 9.2–9.7; the 2′ OH has a far higher p*K*_a_ of ∼13) [17, 31, 32] may play a role in facilitating phosphodiester backbone cleavage next to guanosine. However, we observed no difference between the spectra from CAD of (M − 7H)^7−^ ions of RNA **1** electrosprayed from solutions at pH 8.0, 9.5, and 10.5 (Supplementary Fig. S2) recorded under otherwise the same conditions, from which we conclude that the guanosine bases are not deprotonated in the gaseous RNA ions studied here.

### Effect of site-specific deaza modifications

Preferential cleavage of the phosphodiester backbone next to adenosine in (M + *n*H)^*n*+^ ions of RNA has been shown to result from N3 protonation of adenosine bases and their interactions with uncharged phosphodiester moieties on their 5′ side [10]. Assuming that a similar structure motif involving N3 of guanosine leads to preferential cleavage of the phosphodiester backbone next to guanosine in (M − *n*H)^*n*−^ ions of RNA, we studied RNA **2** with c^3^G at position 5 (Table 1) and compared the data with those of RNA **1** (G at position 5) and RNA **3** (c^1^G at position 5).

**Figure 4.**
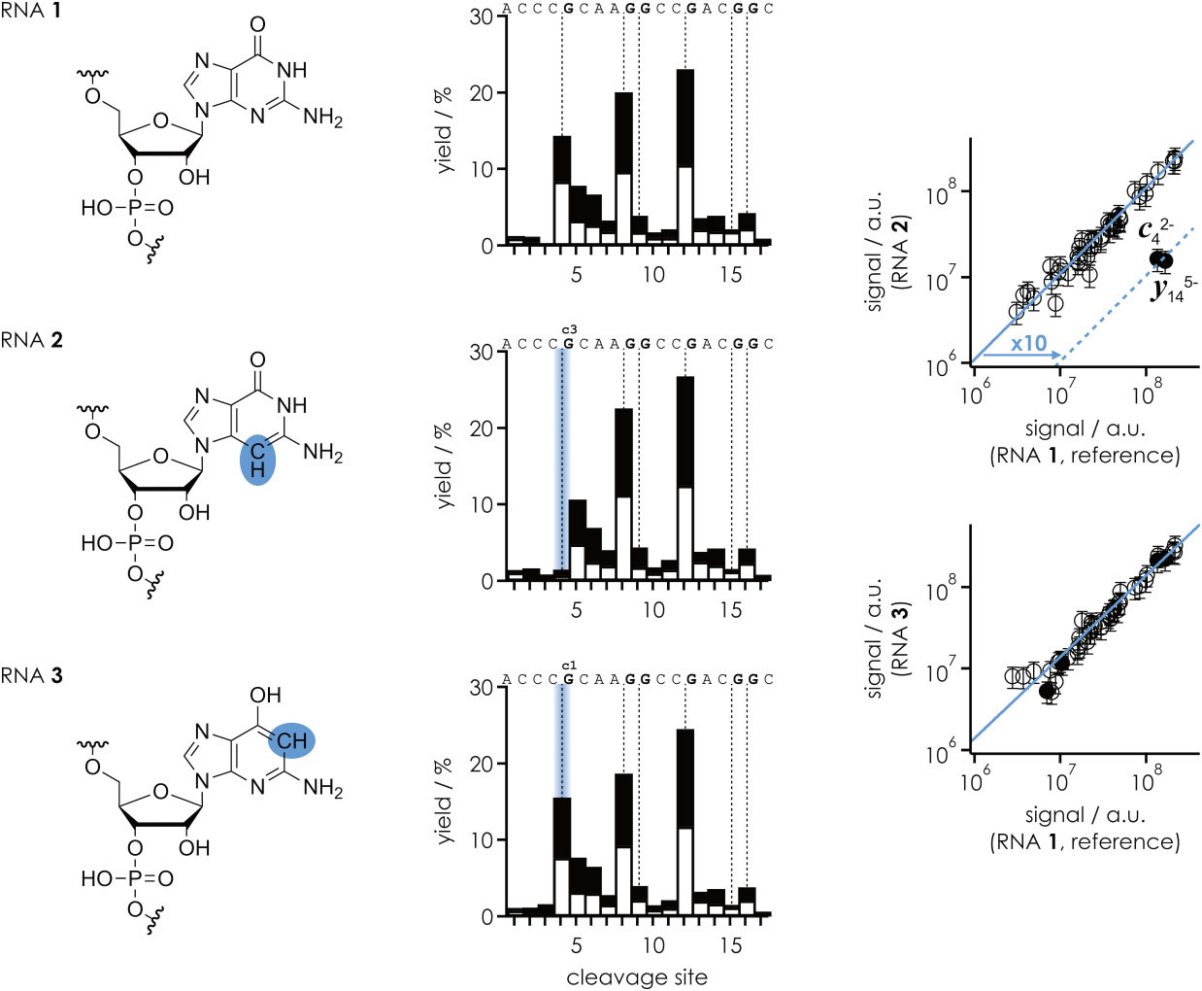
Site-specific yield of *c* (filled bars) and *y* (open bars) fragments (including those that showed nucleobase loss and relative to all *c* and *y* fragments) from CAD of (M − 7H)^7−^ ions of RNAs **1**, **2**, and **3** using a laboratory frame collision energy of 35.0 eV; cleavage sites 5′ of guanosine are highlighted by dashed lines. Correlation plots of the signals of individual *c* and *y* fragments from CAD of (M − 7H)^7−^ ions RNA **2** (c^3^G at position 5) and RNA **3** (c^1^G at position 5) versus those of the unmodified reference RNA **1** (G at position 5) highlight the fragments whose abundance is significantly affected by deaza modification (*c*_4_^2−^ and *y*_14_^5−^ from cleavage on the 5′ side of c^3^G at position 5).

The site-specific yield of *c* and *y* fragments was very similar for RNAs **1**, **2**, and **3** except at site 4, where the yield was ∼15% for RNAs **1** and **3** but only ∼1.5% for RNA **2** (Fig. 4). The ∼10-fold reduction in the abundance of *c*_4_^2−^ and *y*_14_^5−^ fragments from cleavage on the 5′ side of c^3^G at position 5 in RNA **2** when compared to RNA **1** (G) and **3** (c^1^G) is also apparent in the correlation plots in Fig. 4, where the *c* and *y* fragments are resolved by charge and whether or not they show nucleobase loss. These data clearly show that N3 of guanosine must be involved in the interactions that facilitate phosphodiester backbone cleavage and that N1 does not play a role. Moreover, the fact that the data for RNA **1** (G) and **3** (c^1^G) were virtually the same is further evidence against deprotonation of guanosine bases in the (M − *n*H)^*n*−^ ions, since the deprotonation site of G is N1.

### Effect of site-specific removal or methylation of the exocyclic amino group

The structures of RNA (M − *n*H)^*n*−^ ions in the gas phase are generally unknown. For RNA ions with up to 35 nt and very low net charge (corresponding to 0.11–0.25 charges/nt), ion mobility data indicate a substantial compaction of the solution structure after transfer into the gas phase by ESI [33–35], but so far no data have been reported for more highly charged ions. In any case, ion mobility spectrometry can only provide a relatively coarse measure of the compactness of RNA ions without revealing structural features. However, Rodgers and coworkers have determined the gas-phase structures of deprotonated RNA mononucleotides by infrared multiphoton dissociation action spectroscopy, and found nucleobase–phosphate interactions only in the 5′-monophosphates of guanosine [36]. For adenosine, cytidine, and uridine, the nucleobases of the 5′-monophosphates are oriented away from the 5′ phosphate group (*anti* conformation), whereas the *syn* conformation of the 5′-monophosphate of guanosine is stabilized by ionic hydrogen bonding between the negatively charged oxygen atom of the 5′ phosphate and the exocyclic amino group of the guanine moiety.

**Figure 5.**
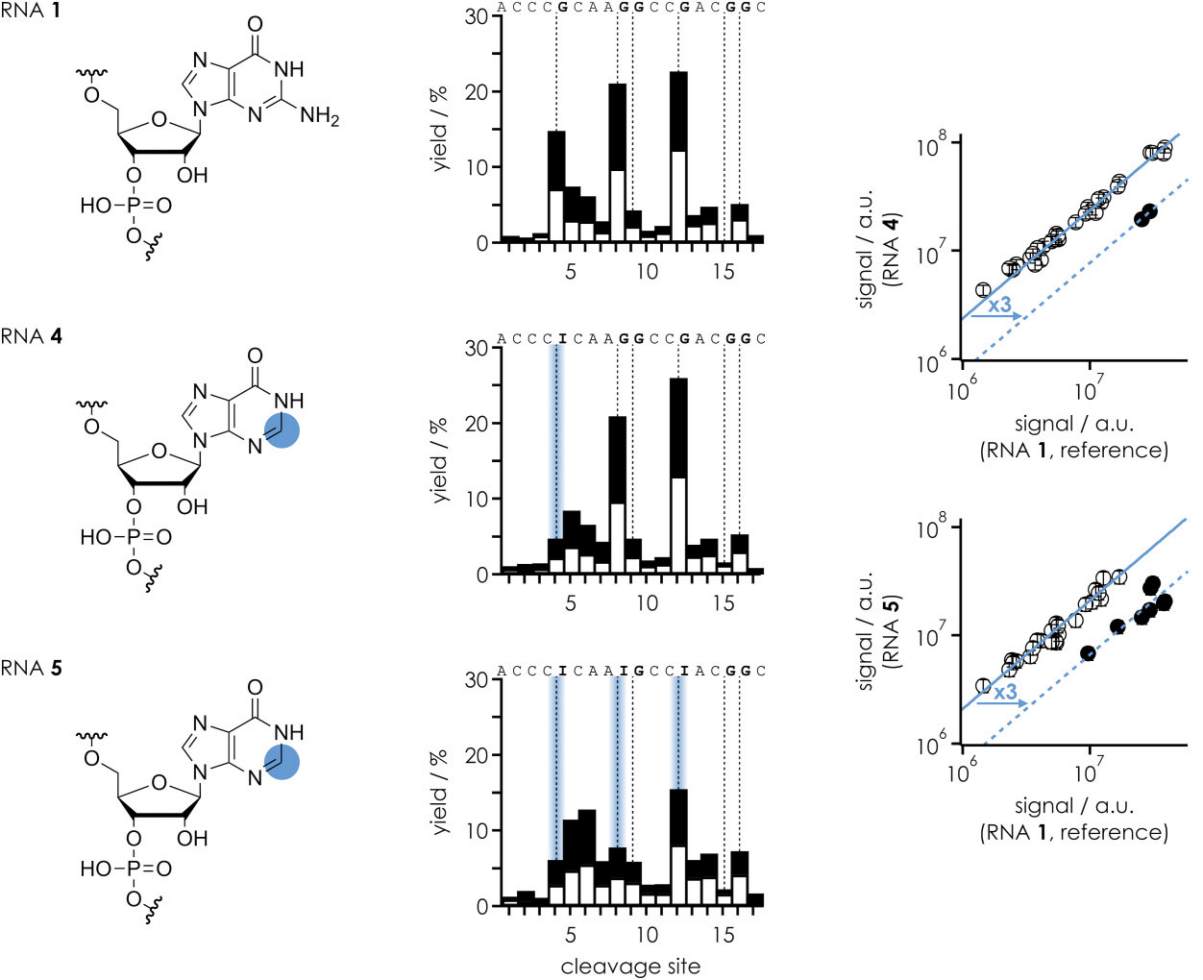
Site-specific yield of *c* (filled bars) and *y* (open bars) fragments (including those that showed nucleobase loss and relative to all *c* and *y* fragments) from CAD of (M − 7H)^7−^ ions of RNAs **1**, **4**, and **5** using a laboratory frame collision energy of 42.0 eV; cleavage sites 5′ of guanosine and inosine are highlighted by dashed lines. Correlation plots of the signals of individual *c* and *y* fragments from CAD of RNA **4** (I at position 5) and RNA **5** (I at positions 5, 9, and 13) versus those of the unmodified reference RNA **1** (G at positions 5, 9, and 13) highlight the fragments whose abundance is significantly affected by removal of the exocyclic amino group.

While our data for (M − *n*H)^*n*−^ ions of RNAs **1**–**3** provide strong evidence for interactions between N3 of guanosine and the phosphodiester moiety on its 5′ side, we thus wondered whether interactions of the exocyclic amino group of guanosine might also play a role in stabilizing the nucleobase–phosphodiester interaction. To address this question, we investigated the 18-nt RNAs **4** and **5** in which individual guanosines were substituted by inosine (I), which lacks the exocyclic amino group of guanosine. As illustrated in Fig. 5, site-specific guanosine to inosine substitution did indeed reduce preferential cleavage on the 5′ side of the corresponding nucleotide residues. Because ions with the same net charge, (M − 7H)^7−^, were used in the CAD experiments in Figs 4 and 5, and RNAs **4** and **5** are both derived from RNA **1**, we can directly compare the effect of guanosine to inosine substitution with that of the deaza modifications. For RNA **4**, the guanosine to inosine substitution at position 5 reduced the effect of guanosine by a factor of ∼3, which is approximately three times less than the reduction observed with c^3^G at the same position (RNA **2**), from which we conclude that the exocyclic amino group is involved in the nucleobase–phosphodiester interaction but that the contribution of N3 to the effect of guanosine is more important than that of the exocyclic amino group.

Furthermore, the data for RNA **5** strongly suggest that the (M − 7H)^7−^ ions of RNAs **1**–**5** with 0.39 charges/nt have largely extended structures in which the guanosine bases can interact only with adjacent phosphodiester moieties, since the reduction from guanosine to inosine substitution is consistently three-fold at all three sites showing a pronounced effect of guanosine, i.e. on the 5′ side of G5, G9, and G13 (Fig. 5).

**Figure 6.**
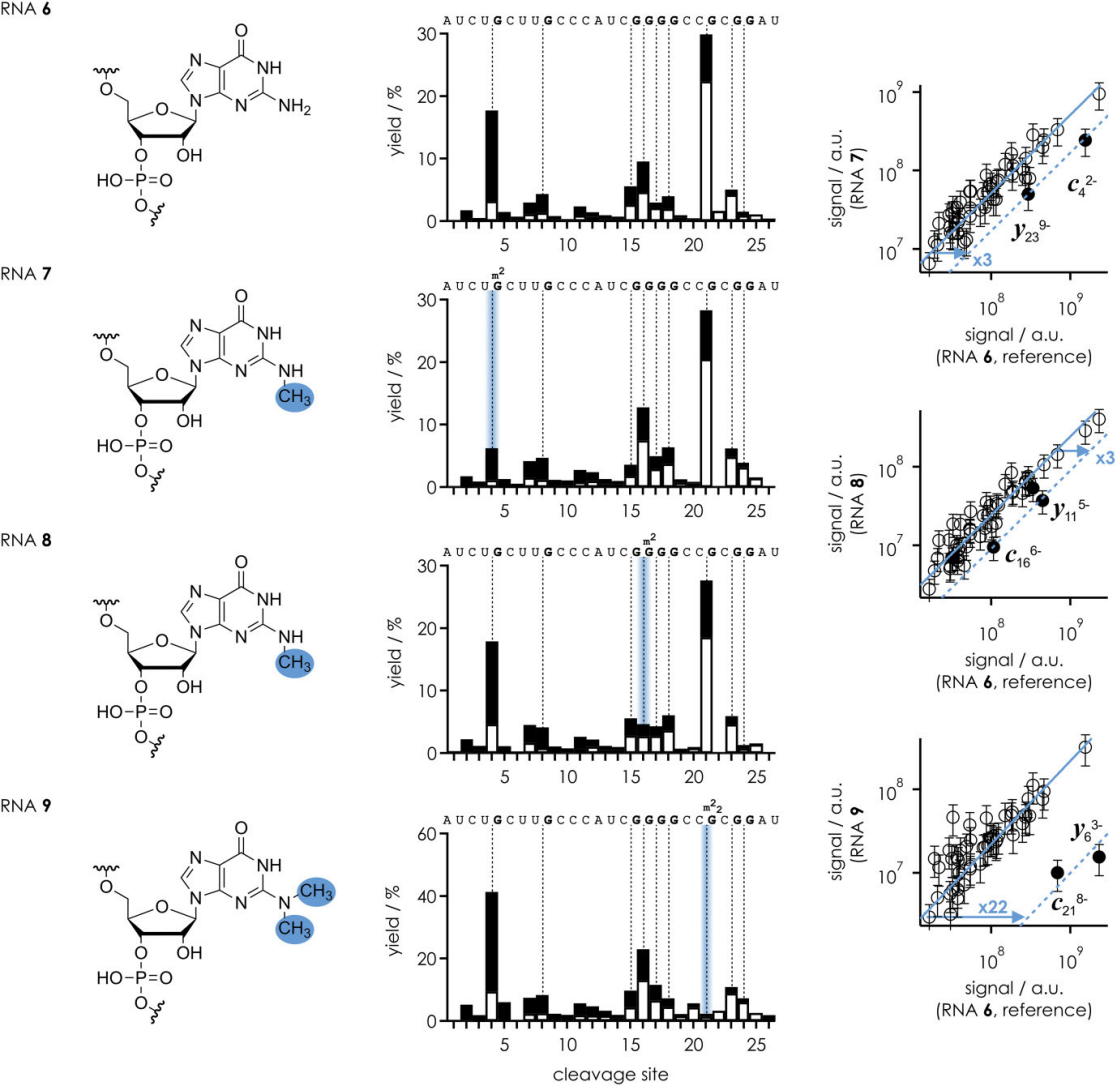
Site-specific yield of *c* (filled bars) and *y* (open bars) fragments (including those that showed nucleobase loss and relative to all *c* and *y* fragments) from CAD of (M − 11H)^11−^ ions of RNAs **6**, **7**, **8**, and **9** using a laboratory frame collision energy of 80.3 eV; cleavage sites 5′ of guanosine are highlighted by dashed lines. Correlation plots of the signals of individual *c* and *y* fragments from CAD of RNA **7** (m^2^G at position 5, shown here with the *N*^2^-methyl group in the *s*-*trans* rotamer), RNA **8** (m^2^G at position 17), and RNA **9** (m^2^_2_G at position 22) versus those of the unmodified reference RNA **6** (G at positions 5, 17, and 22) highlight the fragments whose abundance is significantly affected by mono- or dimethylation of the exocyclic amino group.

Data from CAD of the (M − 11H)^11−^ ions of RNAs **6**–**9** (0.41 charges/nt), without and with site-specific monomethylation (m^2^G) or dimethylation (m^2^_2_G) of the exocyclic amino groups of the guanosine bases at positions 5, 17, and 22, are shown in Fig. 6. Strikingly, monomethylation of the exocyclic amino groups of G5 (RNA **7**) and G17 (RNA **8**) reduced the effect of guanosine by a factor of ∼3, which is the same as that for guanosine to inosine substitution. Although the RNAs used to determine these factors differ in sequence and length, the (M − 7H)^7−^ ions of RNAs **1**–**5** and the (M − 11H)^11−^ ions of RNAs **6**–**9** have very similar and high charge densities (∼0.4 charges/nt), so it is reasonable to assume that the gas-phase structures of the (M − 11H)^11−^ ions of RNAs **6**–**9** are also largely extended. This in turn suggests that monomethylation of the exocyclic amino group disrupts its interaction with the phosphodiester moiety, consistent with the “*s*-*trans*” (or “proximal”) orientation of the *N*^2^-methyl substituent of m^2^G as found in DFT (density functional theory) calculations [37] and structural studies [38–40].

An even larger factor of ∼20 for the reduction of the effect of guanosine was observed with dimethylation of the exocyclic amino group of G22 (RNA **9**), which can be rationalized by steric hindrance. Not only does the dimethylation prevent the interaction of the exocyclic amino group, but its bulkiness apparently also interferes with the interaction of N3 with the phosphodiester moiety. Thus, the “true” effect of guanosine on facilitating backbone cleavage by hydrogen bonding between the nucleobase and the phosphodiester moiety is at least 20-fold.

### Effect of guanosine in shorter RNA

RNA characterization by MS often involves digestion into shorter oligonucleotides followed by liquid chromatography–MS/MS [41]. In addition to *c*- and *y*-type fragments, CAD of shorter RNA also produces *b*-, *d*-, *x*-, and *z*-type fragments [42, 43] that are generally not observed with CAD of RNA >10 nt, and we wondered whether shorter and longer RNA would also behave differently with respect to the effect of guanosine on the formation of *c* and *y* fragments. To address this question, we studied (M − *n*H)^*n*−^ ions of the 8-nt RNAs **10**–**12** with *n* = 2–5 (corresponding to 0.25–0.625 charges/nt). From the data for RNAs **1** and **6** (Fig. 1), which showed a maximum overall effect of guanosine at ∼0.45 and ∼0.4 charges/nt, respectively, we expected a maximum overall effect of guanosine for the (M − 3H)^3−^ (0.375 charges/nt) or (M − 4H)^4−^ (0.5 charges/nt) ions of RNA **10**. Surprisingly, we observed the highest overall effect of guanosine for *n* = 2 (49%) and *n* = 5 (41%), whereas the values for *n* = 3 (35%) and *n* = 4 (26%) were close to the 29% calculated for random cleavage (two sites on the 5′ side of G, seven sites in total) (Supplementary Fig. S3). In addition, the maximum overall effect of guanosine was smaller for RNA **10** (49% at *n* = 2) than for RNAs **1** (75% at *n* = 8) and **6** (81% at *n* = 11). The bar plots for RNA **10** in Supplementary Fig. S3 show that the yield of *c* and *y* fragments was relatively high not only on the 5′ side of G at position 6 (cleavage site 5), but also at other cleavage sites, especially sites 4 and 6. However, replacing G at position 6 (RNA **10**) with c^3^G (RNA **11**) in the (M − *n*H)^*n*−^ ions with *n* = 2–5 (c^7^G of RNA **12** had no effect) reduced cleavage only on the 5′ side of G6 (cleavage site 5), as shown in Supplementary Fig. S4 for *n* = 4. This indicates that G at position 6 facilitates cleavage exclusively at site 5 of the (M − *n*H)^*n*−^ ions of the 8-nt RNAs **10** and **12**, and that the high yields of *c* and *y* fragments from other sites result from protons located on phosphodiester moieties on the 5′ side of bases other than guanosine. However, the site-specific yield of *c* and *y* fragments calculated relative to all *c* and *y* fragments is not ideal for quantifying the effect of guanosine in shorter RNA because, due to this normalization, a significant decrease in yield at a given site (e.g. site 5 in Supplementary Fig. S4) causes a noticeable increase in yield at other sites. A much better measure of the effect of replacing G at position 6 with c^3^G is provided by the correlation plots for RNAs **10** and **11** in Supplementary Fig. S5 for *n* = 2–5. For *n* = 2, *c*_5_^1−^ and *y*_3_^1−^ from cleavage at site 5 are approximately two-fold more abundant for RNA **10** (G) compared to RNA **11** (c^3^G), and for *n* = 3, *c*_5_^2−^ and *y*_3_^1−^ are approximately three-fold more abundant for RNA **10** compared to RNA **11**. For *n* = 4, the ratio for *y*_3_^1−^ is ∼5, and for *n* = 5, the ratio for *c*_5_^3−^ and *y*_3_^2−^ is ∼10. Accordingly, the effect of guanosine on phosphodiester backbone cleavage at site 5 increases from ∼2-fold to ∼10-fold as *n* increases from 2 to 5 when compared to c^3^G, consistent with a decreasing number of protons available for cleavage-facilitating interactions of nucleobases other than G6. Nevertheless, for all (M − *n*H)^*n*−^ ions of the 8-nt RNAs **10** and **12**, the effect of guanosine is clearly masked by other cleavage-facilitating interactions, much more so than for the 18-nt and 27-nt RNAs **1**, **3**, and **6** (Fig. 2 and Supplementary Fig. S1). This suggests that backbone protonation on the 5′ side of A, C, and U is more likely in the shorter RNAs **10** and **12** than in the longer RNAs **1**, **3**, and **6**, probably because the shorter RNAs allow the formation of structures that are significantly different from those of the larger RNAs, which is also consistent with the fact that shorter but not longer RNAs dissociate into *b*-, *d*-, *x*-, and *z*-type fragments upon vibrational activation by CAD.

### Proposed interactions of guanosine with the phosphodiester moiety on its 5′ side

**Scheme 2.**
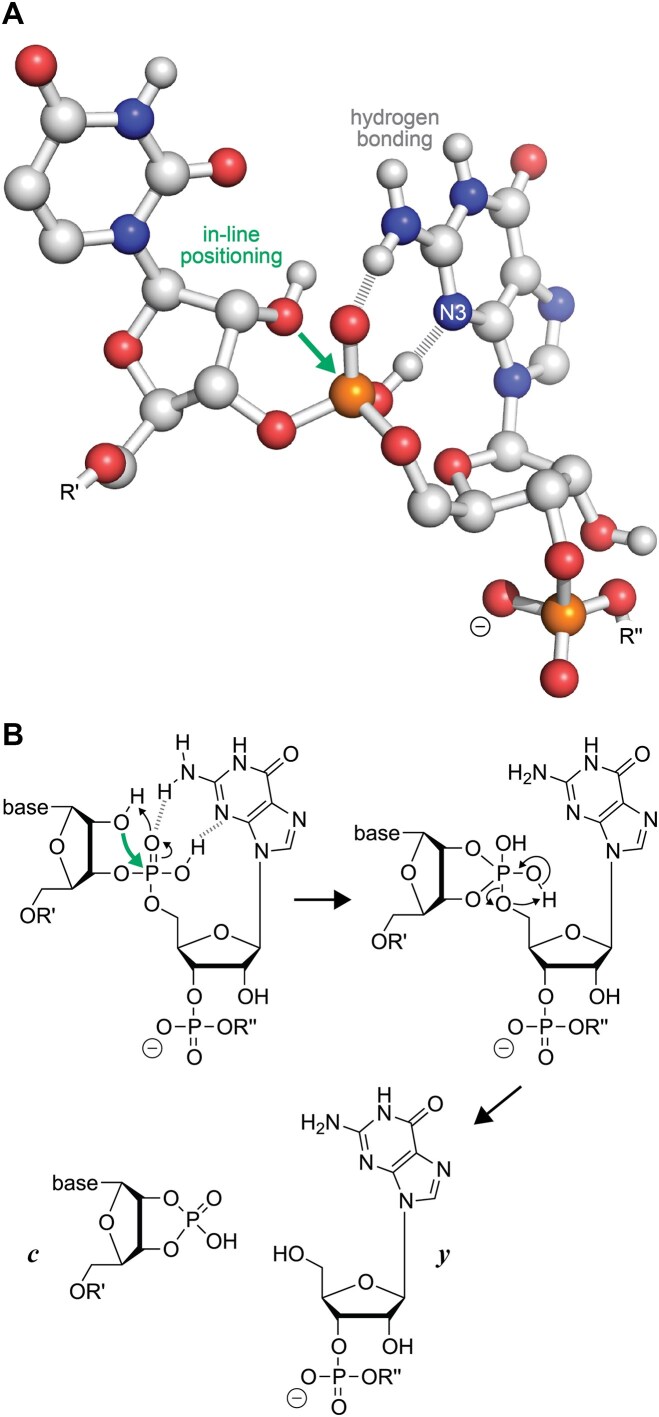
Mechanistic considerations for preferential RNA cleavage 5′ of G in the gas phase. (**A**) Dinucleotide model (UG) illustrating a possible RNA structure in which nucleophilic attack of the ribose 2′ oxygen on the phosphorus (green arrow) is facilitated by hydrogen bonding (dashed lines) between the phosphodiester and guanine moieties and (**B**) proposed mechanism for phosphodiester backbone bond cleavage into *c* and *y* fragments.

The ∼20-fold cleavage-facilitating effect of guanosine observed here raises the question of how exactly do N3 and the exocyclic amino group interact with the phosphodiester moiety in the (M − *n*H)^*n*−^ ions. In the canonical keto–amino form of guanosine, N3 is a proton acceptor, and the exocyclic amino group can be a proton donor. An uncharged phosphodiester moiety can thus form two hydrogen bonds with the guanine moiety, one between N3 and the phosphodiester OH group and the other between the exocyclic amino group and the other nonbridging oxygen. As illustrated in the dinucleotide model in Scheme 2A, these interactions stabilize the proton in the OH–N3 hydrogen bond and also allow for in-line positioning of the 2′ oxygen of the ribose on the 5′ side of guanosine, thereby facilitating phosphodiester backbone bond cleavage into *c* and *y* fragments (Scheme 2B).

In the proposed structure in Scheme 2A, c^1^G (RNA **3**) or c^7^G (RNA **12**) can be substituted for G (RNAs **1** and **10**) without changing the proposed mechanism, consistent with the fact that these modifications did not cause any significant changes in the corresponding CAD spectra (Fig. 4 and Supplementary Fig. S4). In contrast, the substitution of c^3^G (RNAs **2** and **11**) for G (RNAs **1** and **10**) introduces CH in place of the proton acceptor N3, so that the proposed OH–N3 hydrogen bond that stabilizes the proton on the phosphodiester moiety (“β catalysis”) can no longer form, consistent with the up to 10-fold reduction of the effect of guanosine (Fig. 4 and Supplementary Fig. S5). In the proposed mechanism in Scheme 2B, the same proton on the phosphodiester moiety ultimately neutralizes the developing negative charge on the 5′ oxygen (“δ catalysis”). Furthermore, the data for RNAs **4**–**9** with and without site-specific m^2^G and m^2^_2_G modifications or guanosine to inosine substitution strongly suggest that the proposed structure in Scheme 2A, in which the phosphodiester moiety is positioned for in-line attack of the ribose 2′ oxygen (“α catalysis”), is stabilized by the additional hydrogen bond between the exocyclic amino group and the other nonbridging oxygen (Figs 5 and 6). The relatively small reduction in the effect of guanosine by the introduction of m^2^G or inosine (Fig. 5) compared to that of c^3^G suggests that the “catalytic” contribution of the exocyclic amino group is smaller than that of N3. The largest reduction was observed for m^2^_2_G, whose bulkiness prevents the hydrogen bonding interactions of both the exocyclic amino group and N3 with the phosphodiester moiety by steric hindrance (Fig. 6). The proposed structure in Scheme 2 also explains why the yields of *c* and *y* fragments from phosphodiester backbone bond cleavage on the 5′ side of A, C, and U are much lower than those of G. Adenine shares N3 with guanine, and the pyrimidine bases cytosine and uracil have a carbonyl oxygen at the position corresponding to N3 in the purine bases. While both the N3 of A and the carbonyl oxygens of C and U are proton acceptors, all of these bases lack the exocyclic amino group at C2 as proton donor. This means that, unlike G, they can only form a single hydrogen bond and the interactions with the phosphodiester moiety must be much weaker compared to G. Moreover, two hydrogen bonds (G) instead of only one (A, C, U) should allow a more precise in-line positioning of the 2′ oxygen of the ribose for nucleophilic attack.

The penta-coordinated oxyphosphorane in Scheme 2B can in principle represent either an intermediate or a transition state structure [44, 45]. In our previous CAD study of RNA (M + *n*H)^*n*+^ and (M − *n*H)^*n*−^ ions [9], we have discussed evidence for a stepwise reaction involving a penta-coordinated oxyphosphorane “intermediate” for phosphodiester backbone bond cleavage in the gas phase. A key argument was that the charge values of *c* and *y* fragments indicated charge locations according to Coulombic repulsion in extended RNA structures rather than those indicated by preferential backbone cleavage, implying that intramolecular proton transfer must have occurred after nucleophilic attack but before backbone cleavage [9]. For RNA (M + *n*H)^*n*+^ ions, we have reported additional support for this hypothesis by showing that the fragment charge values were the same for RNA without and with site-specific deaza modification that abolished the effect of adenosine [10]. To further test the hypothesis that an intermediate is also involved in phosphodiester backbone cleavage of RNA (M − *n*H)^*n*−^ ions, we analyzed the charge values of *c* and *y* fragments for the 18-nt RNAs **1** and **2** and the 27-nt RNAs **6** and **9**. As shown in Supplementary Fig. S6, the m^2^_2_G and c^3^G modifications, which caused ∼20- and ∼10-fold reduction in the effect of guanosine, respectively, did not significantly alter the charge values of *c* and *y* fragments, consistent with intramolecular proton redistribution according to Coulombic repulsion in extended structures after nucleophilic attack. According to calculations by York and coworkers [46], penta-coordinated oxyphosphoranes are much less acidic than acyclic phosphodiesters both in the gas phase and in solution, so the proton on the penta-coordinated oxyphosphorane should still be available for neutralization of the developing negative charge on the 5′ oxygen after intramolecular proton redistribution. However, because the proton is directly involved in the cleavage step, we can only conclude that intramolecular proton transfer must have occurred after nucleophilic attack and before or during backbone cleavage, so the data in Supplementary Fig. S6 do not allow us to distinguish between a transition and an intermediate state of the penta-coordinated oxyphosphorane in CAD of (M − *n*H)^*n*−^ ions.

### RNA hydrolysis in basic solutions

**Figure 7.**
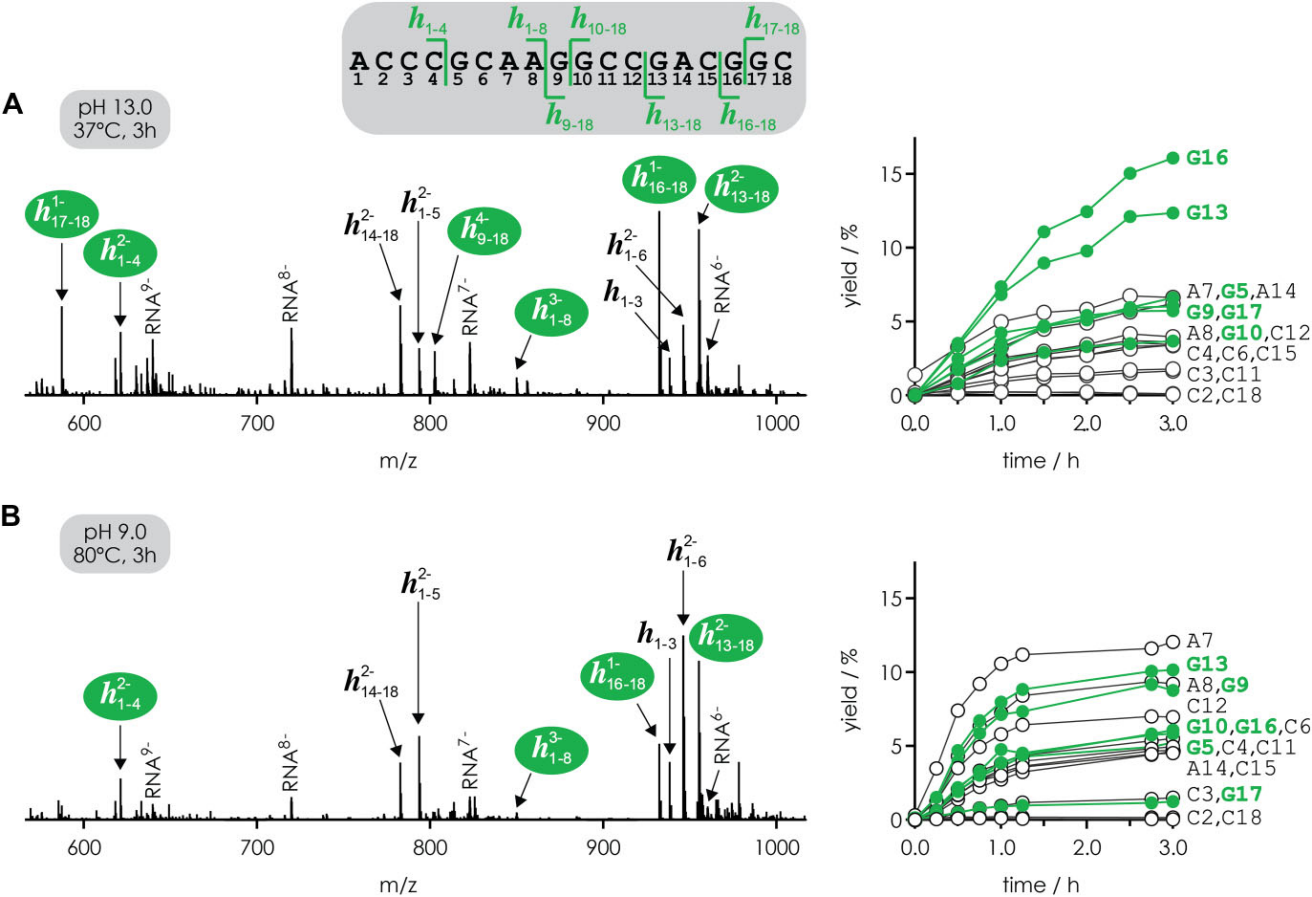
MS analysis of products from hydrolysis of RNA **1** in aqueous solutions at high and near-neutral pH. (**A**) A representative section of the mass spectrum of products *h*_*i*-*j*_, where the indices *i* and *j* indicate the numbering of residues in the original sequence, from hydrolysis at pH 13.0 (100 mM piperidine) and 37°C after 3 h shows that the most abundant hydrolysis products were from backbone cleavage on the 5′ side of G13 (*h*_13-18_) and G16 (*h*_16-18_); the time-resolved yields of hydrolysis products *h*_*i*-*j*_ from backbone cleavage on the 5′ side of G are highlighted in green. (**B**) For hydrolysis at pH 9.0 (4 mM piperidine) and 80°C after 3 h (bottom), no preference for backbone cleavage on the 5′ side of G was observed. For clarity, only abundant signals are labeled in the spectra.

To assess whether the effect of guanosine on RNA backbone cleavage observed for (M − *n*H)^*n*−^ ions reflects an intrinsic property of RNA or is merely an artifact resulting from the gas-phase environment, we studied the hydrolysis of RNA **1** in aqueous solution at highly basic and near-neutral pH. Figure 7 shows representative sections of mass spectra of the products from hydrolysis of RNA **1** at pH 13.0 and 37°C and pH 9.0 and 80°C after 3 h, and the hydrolysis reaction kinetics for 0–3 h. At pH 13.0, the most abundant hydrolysis products were *h*_13-18_ and *h*_16-18_ from phosphodiester backbone cleavage on the 5′ site of G13 and G16, respectively; products from backbone cleavage next to residues other than G were the least abundant at all hydrolysis times studied. At pH 9.0, no preference for backbone cleavage on the 5′ side of G was observed. These data demonstrate that the effect of guanosine on phosphodiester backbone cleavage can be observed not only in the gas phase but also in solution, from which we conclude that it is based on an intrinsic property of RNA. The effect is more pronounced at higher pH in solution and at higher (M − *n*H)^*n*−^ ion net charge in the gas phase, consistent with our hypothesis that guanosine bases hold protons in place on the phosphodiester moieties on their 5′ side such that phosphodiester moieties adjacent to bases other than guanosine are preferentially deprotonated. Although we did not find any evidence for deprotonation of N1 in the gaseous (M − *n*H)^*n*−^ ions, N1 of the guanosine base is deprotonated in solutions at pH 13.0 (p*K*_a_ of N1: 9.2–9.7), which could contribute to the observed cleavage preference in solution by strengthening hydrogen bonding between N3 and the phosphodiester moiety.

## Conclusions

The gas-phase data reported here reveal the intrinsic preference of guanosine to orient its nucleobase in *syn* conformation in RNA (M − *n*H)^*n*−^ ions and form two hydrogen bonds with the phosphodiester moiety on its 5′ side in a structure that positions the adjacent 2′ oxygen for nucleophilic attack, thereby enabling facile cleavage of the phosphodiester backbone bond into *c* and *y* fragments upon vibrational activation by CAD. No such preference was observed for adenosine, cytosine, and uridine. A similar effect of guanosine facilitating the hydrolysis of the phosphodiester on its 5′ side was observed in solutions at high pH, in agreement with data from a kinetic study in solutions at pH ∼12.0–14.5 by Li and Breaker, who found that the rate constants for phosphodiester backbone bond cleavage between adenosine and guanosine on its 3′ side (“ApG”) were ∼2-fold higher than those between two adenosines (“ApA”) [17]. The preferential cleavage of phosphodiester backbone bonds on the 5′ side of guanosines, as observed in both basic solutions and for gaseous (M − *n*H)^*n*−^ ions, suggests an intrinsic structural preference that may have served as an (initial) minimal conformational (dinucleotide) module to evolve catalytic activity in more complex three-dimensional RNA arrangements, in particular of ribozymes that cleave their phosphodiester backbone. In support of this hypothesis, a statistical analysis of RNA structures from the Protein Data Bank (https://www.rcsb.org/) revealed a significant number of structures in which the phosphorus atoms of phosphodiester moieties were in close proximity to both N3 and the exocyclic amino group of guanosine [47]. The effect of guanosine involves several intricate factors, among them the RNA sequence and the (M − *n*H)^*n*−^ ion net charge or the solution pH, all of which affect RNA structure and thus the intramolecular interactions of individual guanosines. In this setting, depending on local charge distributions and hydrogen bond interactions, different guanosines in a given sequence can exhibit varying degrees of this effect.

For characterization or identification of RNA primary structure with high sequence coverage by top-down MS, this study confirms previous recommendations [48] to use RNA (M − *n*H)^*n*−^ ions with lower net charge (<0.4 charges/nt) for CAD, which promotes the formation of *c* and *y* fragments while minimizing preferential cleavage next to guanosine. Further, care should be taken when using top-down MS for label-free, direct localization and relative quantification of RNA nucleobase methylations [49] that affect phosphodiester backbone cleavage by CAD, i.e. m^2^G and m^2^_2_G.

## Supplementary Material

gkaf494_Supplemental_File

## Data Availability

The data underlying this article are available in the article and in its online supplementary material.
