# Supplementary material for: The intrinsic preference of guanosine bases for cleavage-facilitating interactions with phosphodiester moieties in RNA anions revealed by base modifications and mass spectrometry
Source: Nucleic Acids Res. 2025 Jun 16;53(11):gkaf494. doi: 10.1093/nar/gkaf494 (PMC12168075; doi:10.1093/nar/gkaf494)
Supplement: gkaf494_Supplemental_File [file gkaf494_supplemental_file.pdf]

The intrinsic preference of guanosine bases for cleavage-facilitating interactions with phosphodiester moieties in RNA anions revealed by base modifications and mass spectrometry

Anna Ploner, Christoph Mitteregger, Heidelinde Glasner, Raphael Bereiter, Ronald Micura, and Kathrin Breuker*

Institute of Organic Chemistry and Center for Molecular Biosciences Innsbruck (CMBI), University of Innsbruck, Innrain 80-82, 6020 Innsbruck, Austria

* To whom correspondence should be addressed. Tel: +43 512 507 57740; Fax: +43 512 507 57799; Email: kathrin.breuker@uibk.ac.at

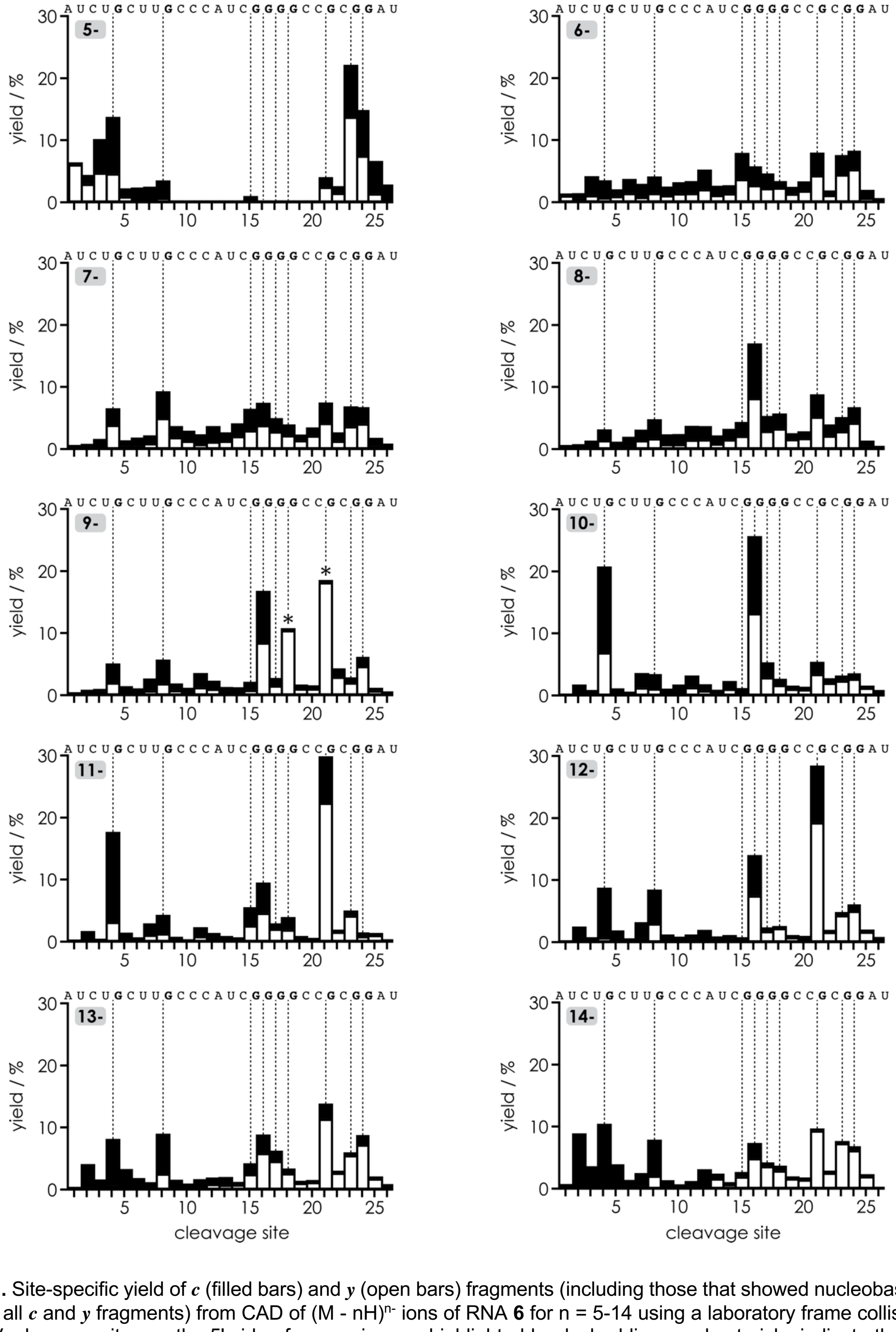

Figure S1. Site-specific yield of c (filled bars) and y (open bars) fragments (including those that showed nucleobase loss and relative to all c and y fragments) from CAD of $(M - nH)^{n-}$ ions of RNA **6** for n = 5-14 using a laboratory frame collision energy of 80.3 eV; cleavage sites on the 5' side of guanosine are highlighted by dashed lines and asterisks indicate that yields for c_{18}^{6-} and c_{21}^{7-} fragments could not be determined due to signal overlap with signals from undissociated RNA ions.

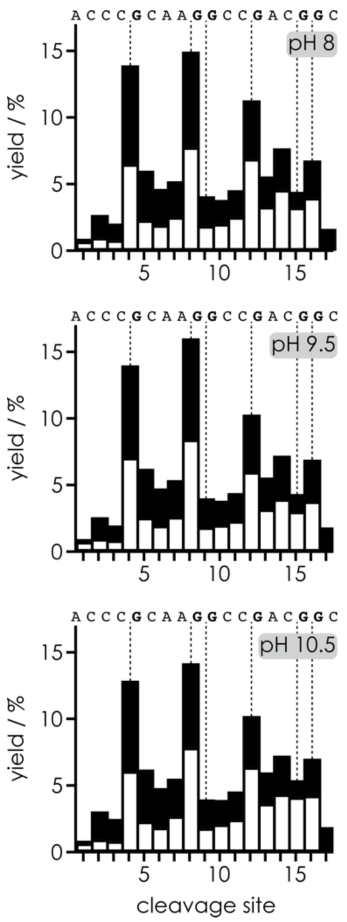

Figure S2. Site-specific yield of c (filled bars) and y (open bars) fragments (including those that showed nucleobase loss and relative to all c and y fragments) from CAD of $(M - 7H)^{7-}$ ions of RNA **1** electrosprayed from solutions at pH 8.0, 9.5, and 10.5, and using a laboratory frame collision energy of 35.0 eV; cleavage sites on the 5' side of guanosine are highlighted by dashed lines.

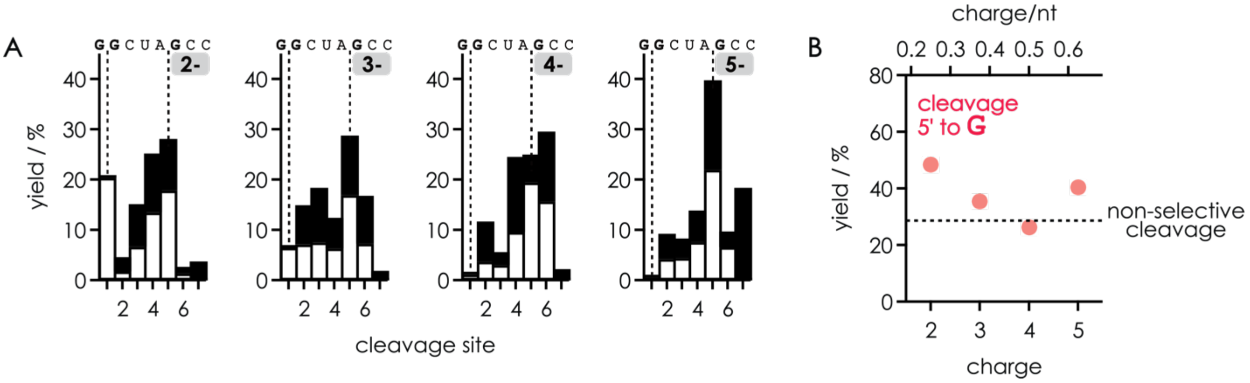

Figure S3. A) Site-specific yield of c (filled bars) and y (open bars) fragments (including those that showed nucleobase loss and relative to all c and y fragments) from CAD of $(M - nH)^{n-}$ ions of RNA **10** with n = 2-5 using laboratory frame collision energies of 40.0, 36.0, 28.0, and 21.0 eV, respectively; cleavage sites on the 5' side of guanosine are highlighted by dashed lines. B) For the 8 nt RNA **10**, the yield of c and y fragments from phosphodiester backbone cleavage on the 5' side of all guanosine residues relative to all c and y fragments did not show a maximum at ~0.45 charges/nt, in contrast to the 18 nt and 27 nt RNAs **1** and **6** (see Figure 1).

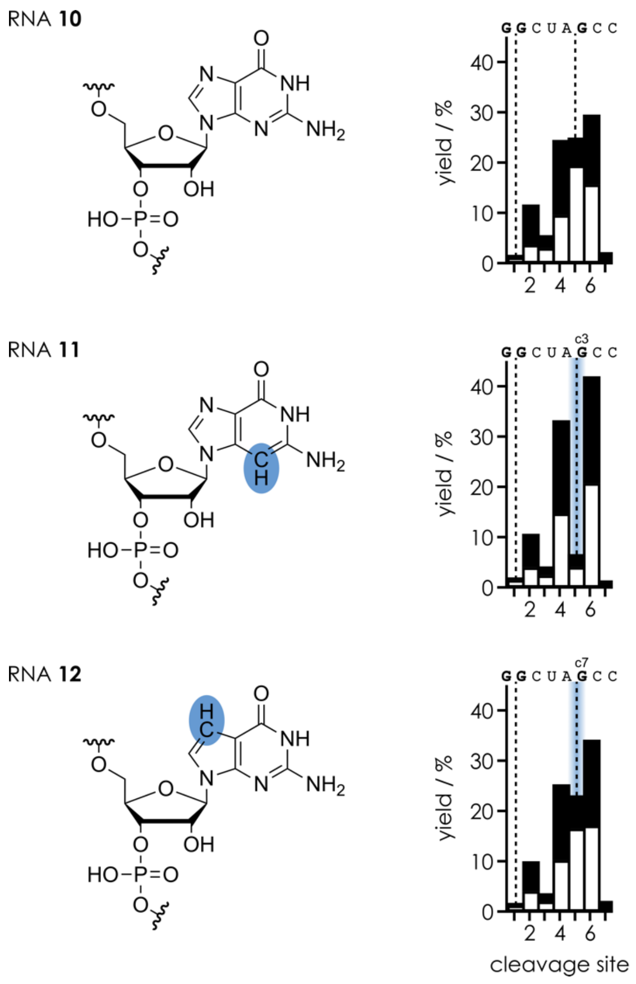

Figure S4. Site-specific yield of c (filled bars) and y (open bars) fragments (including those that showed nucleobase loss and relative to all c and y fragments) from CAD of $(M - 4H)^{4-}$ ions of RNAs **10**, **11**, and **12** using a laboratory frame collision energy of 28.0 eV, cleavage sites 5' of guanosine are highlighted by dashed lines.

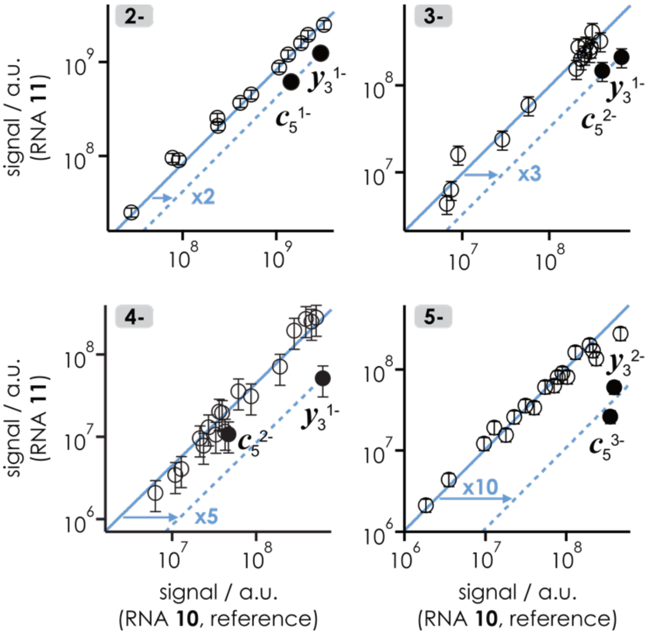

Figure S5. Correlation plots of the signals of individual c and y fragments from CAD of $(M - nH)^{n-}$ ions of RNA **11** (c^3G at position 6) versus those of the unmodified reference RNA **10** (G at position 6) for n = 2-5 highlight the fragments whose abundance is significantly affected by deaza modification (c_5 and y_3 from cleavage on the 5' side of c^3G at position 6).

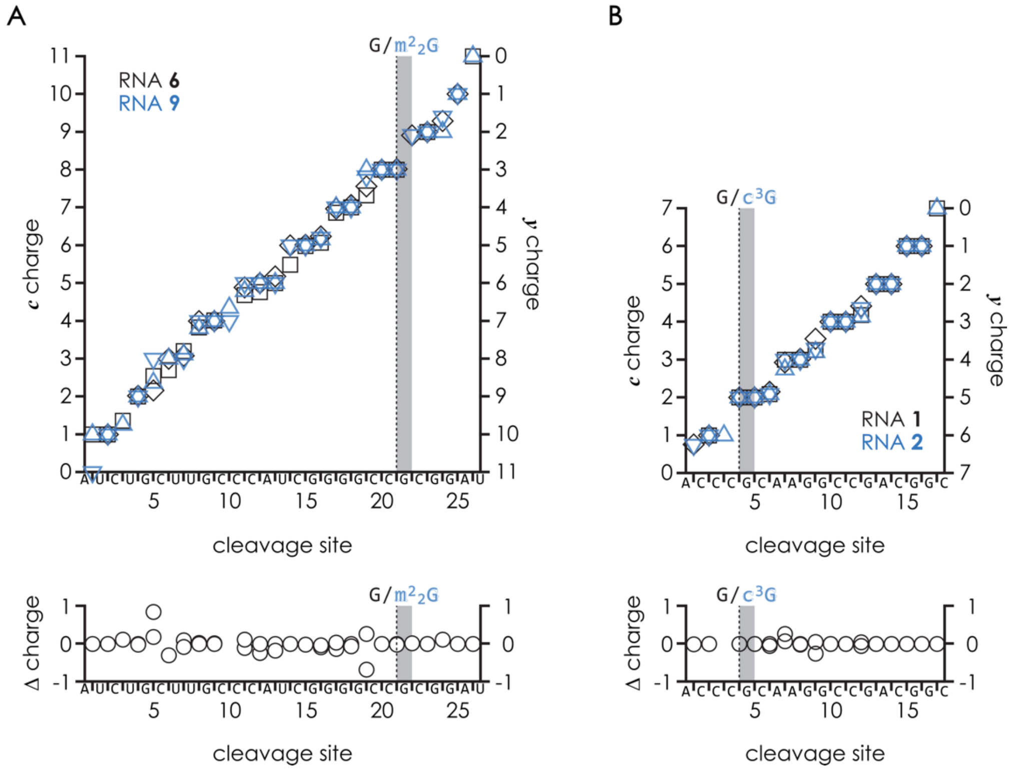

Figure S6. Average charge of *c* (upward triangles and squares, left axes) and *y* (downward triangles and diamonds, right axes) fragments from CAD of A) (M - 11H)$^{11-}$ ions of RNAs **6** (G at position 22, black) and **9** ($m^2{}_2G$ at position 22, blue) using a laboratory frame collision energy of 80.3 eV and B) (M - 7H)$^{7-}$ ions of RNAs **1** (G at position 5, black) and **2** (c^3G at position 5, blue) at 35.0 eV; lower panels show the differences in charge between fragments of unmodified and modified RNAs.
